# The Spider: a visual, multisystemic symptom impact questionnaire for people with hypermobility-related disorders—validation in adults

**DOI:** 10.1007/s10067-024-07071-7

**Published:** 2024-07-31

**Authors:** E. R. Ewer, R. De Pauw, H. Kazkazk, N. Ninis, P. Rowe, J. V. Simmonds, I. De Wandele

**Affiliations:** 1 UCL Great Ormond Street Institute of Child Health, London, UK; 2 Department of Epidemiology, Sciensano, Brussels, Belgium; 3 Department of Rehabilitation Sciences, Ghent University, Ghent, Belgium; 4 University College London Hospital NHS Trust, London, UK; 5 St Mary’s Hospital, Imperial College Healthcare NHS Trust, London, UK; 6 Johns Hopkins University, Baltimore, USA; 7 London Hypermobility Unit, Central Health Physiotherapy, London, UK; 8 Centre for Medical Genetics, Ghent University Hospital, Ghent, Belgium

**Keywords:** Ehlers-Danlos syndrome, Hypermobility, Multisystemic comorbidities, Screening tool, Spider questionnaire

## Abstract

**Introduction:**

Hypermobility spectrum disorders (HSD) and hypermobile Ehlers-Danlos syndrome (hEDS) are often accompanied by varied and complex multisystemic comorbid symptoms/conditions. The Spider questionnaire was developed to evaluate the presence and impact of eight common multisystemic comorbidities. Thirty-one questions across eight symptom domains assess neuromusculoskeletal, pain, fatigue, cardiac dysautonomia, urogenital, gastrointestinal, anxiety, and depression symptoms. This study aimed to evaluate the Spider’s construct validity in adults.

**Method:**

A cross-sectional observational study was conducted over four stages. Three international patient charities aided recruitment of participants through social media and website advertisements. Adults aged 18 to 65 years, with and without HSD/hEDS, were invited to participate. Validated, frequently used comparator questionnaires were used to establish convergent validity of Spider symptom domains. A control group was recruited for known-group validity analysis. Participants answered each Spider domain and the corresponding comparator questionnaire via surveys hosted by REDCap. Anonymous data were analysed using SPSS. Convergent validity was assessed through Spearman’s correlational analysis and known-group validity through Mann–Whitney *U* analysis.

**Results:**

A total of 11,151 participants were recruited across the four stages. Statistically significant, moderate-to-strong correlations were found between all Spider domains and their comparators (*p* < 0.001, *r* = 0.63 to 0.80). Known-group validity analysis showed statistically significant differences (*p* < 0.001) between the hypermobile and control groups in all eight domains.

**Conclusions:**

Convergent and known-group validity of the Spider was established with adults. These results suggest the Spider can measure the presence and impact of multisystemic comorbid symptoms/conditions in adults with HSD/hEDS, providing a tool which guides multidisciplinary management.

**Table:** 

**Key Points** • *The Spider questionnaire is a novel tool assessing the presence and impact of the multisystemic comorbid symptoms/conditions associated with HSD/hEDS.* • *Convergent and known-group validity of the Spider questionnaire was established in adults aged 18 to 65.* • *This tool provides a quick and easy method to visualise the symptom profile of those with HSD/hEDS to guide symptom management.*

## Introduction

Joint hypermobility (JH) is defined as an increased active and passive range of joint motion, either locally or generally. The presence of JH is thought to be influenced by age, sex, and ethnicity and is often asymptomatic [1]. However, some adults with JH can experience debilitating symptoms across multiple body systems. Symptomatic hypermobility (SH) has been described using varying terminology, but since 2017, diagnostic criteria and guidelines created by the International Ehlers-Danlos Syndrome (EDS) consortium advise use of the terms ‘hypermobility spectrum disorder’ (HSD) and ‘hypermobile EDS’ (hEDS) [2]. Whilst these two conditions are differentiated during diagnosis, they present with similar and often complex multisystemic symptom profiles. Around 90% of patients with hEDS report generalised body or soft tissue pain, 78% report neuropathic pain [3], and up to 89% of individuals with HSD report chronic musculoskeletal pain [4]. Joint instability is common, with 78% of people with HSD/hEDS reporting dislocations [3]. Chronic fatigue affects 77% of individuals with HSD/hEDS [5]. In both patient groups, symptoms often extend beyond the musculoskeletal system. Gastrointestinal issues are prevalent, with symptoms such as abdominal pain, constipation, and diarrhoea reported by 47–73%, of people with HSD/hEDS, compared to 9–27% of individuals without HSD/hEDS [6]. Orthostatic intolerance, including postural orthostatic tachycardia syndrome (POTS), affects 60 to 80% of patients with HSD/hEDS, and around 25% of patients with POTS are also diagnosed with HSD/hEDS [7]. Urinary incontinence has been reported in 68–84% of women with HSD/hEDS compared to 25–45% of women in the general population, and genital pain/discomfort is reported by 60% of women with HSD [8]. Research suggests that approximately 70% of individuals with HSD/hEDS have some type of anxiety disorder and have a 4.1 to 4.4 times greater probability of being affected by depression [9] and anxiety [10], respectively, compared to the general population.

Whilst these symptoms are commonly reported by the HSD/hEDS community, the presence and impact of each symptom/condition vary greatly between individuals and throughout their lifetime, leading to a population of adults with diverse and complex symptom profiles [11]. The impact poor functioning of one body system may have on other systems is an important consideration and has been explored in some areas [11] [12] [13] [14]. It is becoming evident that the presence of these comorbid conditions influences the individual’s disease severity and level of disability [11]. Multisystemic symptoms can greatly impact quality of life (QoL) and people with HSD/hEDS often report poor health-related QoL (HRQoL) [15–18]. Those with multiple comorbid symptoms may have a worse prognosis and health status, particularly if symptoms are overlooked and left unmanaged [19, 20].

Whilst the exact prevalence of HSD/hEDS is difficult to quantify due to varying terminology and diagnostic criteria, it is thought to affect around 1 in 500 patients in the UK [21]. This suggests HSD/hEDS is not as rare as previously thought, but a common pathology which burdens healthcare services [21]. Patients with HSD/hEDS frequently require healthcare services; in the UK, 39% of patients in pain management and 37% of individuals treated by rheumatologists have HSD/hEDS [22]. Despite these large numbers of patients with HSD/hEDS seeking care, evidence suggests that clinician knowledge around the associated multisystem comorbidities is lacking [23]. Patients may not associate systemic symptoms with their HSD/hEDS, so unless clinicians ask about these symptoms during appointments, they may be missed and remain unmanaged [24]. Awareness of the presence and impact of these symptoms helps ensure care is holistic and patients receive appropriate multidisciplinary management, reducing functional impairment and disability.

To identify each individual’s symptom profile and help direct care, an international multidisciplinary group of researchers and clinicians developed the ‘Spider’ questionnaire. The Spider addresses the eight main symptom domains commonly associated with HSD/hEDS. These include pain, fatigue, neuromusculoskeletal symptoms, cardiac dysautonomia, urogenital and gastrointestinal symptoms, anxiety, and depression (supplementary material). This concise screening tool allows researchers, clinicians, and patients to assess the presence and impact of symptoms, easily identify areas of need, and guide management. The tool was validated for use in adolescents in 2024 [25] with acceptable convergent and known-group validity. The present study aims to establish the construct validity of the Spider domains in an adult population.

## Materials and methods

Ethical approval was granted by the University College London Research Ethics Committee (19,629/002). A series of studies were undertaken to examine the construct validity (convergent and known-group validity) of the Spider domains with adults aged 18 to 65. As large numbers of questions were required for the validation of each domain, the validation was completed in four stages between July 2023 and November 2023 to reduce participant fatigue. A cross-sectional questionnaire research design was used. Each stage analysed the validity of one, two, or three Spider domains to evenly distribute the number of questions across the stages. The most appropriate validated comparator patient-rated outcome measures (PROMs) were used to assess the Spider’s convergent validity, chosen based on their concepts and psychometric properties.

- Stage 1: The Spider neuromusculoskeletal, fatigue, and gastrointestinal domains were validated using select questions of the Bristol Impact of Hypermobility questionnaire (BIoH) [26], the Checklist for Individual Strength (CIS) [27], Pittsburgh Sleep Quality Index (PSQI) [28], and the Gastrointestinal Symptom Rating Scale (GSRS) [29] as comparators retrospectively.
- Stage 2: The Spider anxiety and depression domains were validated using the General Anxiety Disorder (GAD-7) [30] and Patient Health Questionnaire (PHQ-9) [31] as comparators respectively. The Hospital Anxiety and Depression Scale (HADS) [32] was also used with 300 participants due to funding limitations.
- Stage 3: The Spider cardiac dysautonomia domain was validated using select questions from the COMPASS-31 [33]. The Spider pain domain was validated using the Multidimensional Pain Inventory (MPI) [34] Sects. 1 and 3 and select questions from the BIoH [26].
- Stage 4: The Spider urogenital domain was validated using the symptoms of Lower Urinary tract dysfunction Research Network (LURN) Symptom Index-29 (LURN SI-29) [35] as a comparator.

### Participants

For all stages, participants were recruited using self-selection and snowball sampling. Three hypermobility charities (Hypermobility Syndromes Association, Ehlers-Danlos Support UK, and the Ehlers-Danlos Society) aided recruitment by posting study advertisements on their website, social media pages, or newsletters. Participants were asked to self-identify guided by inclusion criteria (Table 1) and encouraged to ask hypermobile and non-hypermobile family members or friends to participate. It was made clear in study advertisements that a healthy control group (no hypermobility) was also required for comparison. Participants were included if they had diagnoses of HSD and hEDS or had symptomatic hypermobility (SH), referred to collectively as the ‘SH-group’. The inclusion of those with SH was to ensure individuals who have comorbid symptoms and were awaiting official diagnoses of HSD/hEDS were not excluded from participating. The age range was set at 18 to 65, as after the age of 65, people are considered ‘elderly’ [36] and it becomes more difficult to determine if symptoms are associated with aging or HSD/hEDS. Whilst researchers recognise people do not biologically age at the same rate, as this research was conducted virtually with no means to assess frailty, an age limit of 65 was agreed after researching prevalence of multisystemic symptoms in those aged 65 and above [37–41].

**Table 1:** Inclusion and exclusion criteria

Inclusion criteria	Exclusion criteria
Aged between 18 and 65 Diagnosis of HSD/hEDS Diagnosis of symptomatic hypermobility No symptomatic hypermobility (known-group) Able to understand and communicate in English	Younger than 18 or older than 65 Unrelated neurological comorbidities (for example cerebral palsy) Musculoskeletal comorbidities unrelated to hypermobility (for example a traumatic injury) Unrelated rheumatological comorbidities (for example rheumatoid arthritis) GHJ with no symptoms

Key: *GHJ* generalised joint hypermobility, *HSD* hypermobility spectrum disorder, *hEDS* hypermobile Ehlers-Danlos syndrome

### Procedure

If participants self-identified as meeting the inclusion criteria for the SH or control group, they accessed the study questionnaires via an online survey hosting platform REDCap^©^. They were provided with study information and asked to consent to providing anonymous data. Participants provided demographic information (including age, sex assigned at birth, gender, ethnicity, location, diagnosis, and the clinician who provided the diagnosis) and answered the Spider domain and comparator questions. To differentiate between participants with and without SH, participants were asked to choose their diagnosis and who diagnosed them during demographic data collection. The options provided for diagnosis were ‘HSD’, ‘hEDS’, ‘SH’, and ‘I do not have any of the above conditions’. For the control group, the option of ‘not applicable, I do not have hypermobility’ was available for the question ‘professional who gave diagnosis’. Only participants choosing ‘I do not have any of the above conditions’ and ‘not applicable, I do not have hypermobility’ were included in the control group.

### The Spider questionnaire

**Fig. 1:**
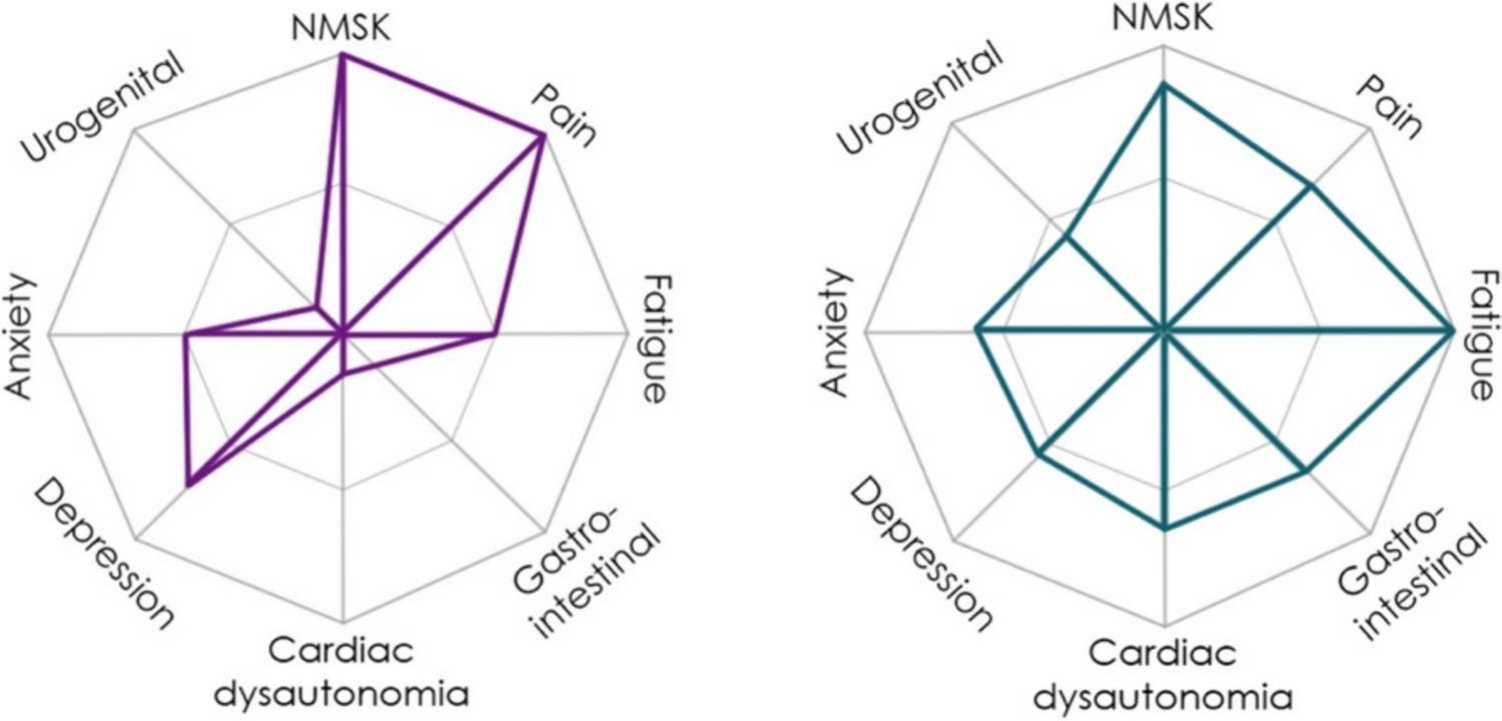
An example of two completed questionnaires’ radar graphs [25]. Descriptive caption: a picture showing two diagrams, shaped like a Spider’s web, with eight symptom domains as described above. Within each diagram are points mapped to each symptom joined together by lines. The closer to the edge the point is, the larger the impact of this symptom for the individual. Each diagram has different points, demonstrating different symptom profiles

The Spider consists of 31 questions across eight symptom domains commonly associated with HSD/hEDS, including pain, fatigue, neuromusculoskeletal symptoms, cardiac dysautonomia, urogenital and gastrointestinal symptoms, and anxiety and depression (supplementary material). A six-point Likert scale is used to score answers, with ‘not present’ and ‘no impact on daily life’ scoring 0, and ‘mild’, ‘moderate’, ‘severe’, and ‘disabling’ impact scoring 25, 50, 75, and 100 respectively. Two questions have alternative scoring (q.19 and q.25) scored between 0 and 100. Each domain is scored by averaging the domain questions total, providing an overall domain percentage, with higher scores indicating higher impact on daily living. A radar graph is produced upon completion of the questionnaire, providing a visual overview of a patient’s symptom profile (Fig. 1). Further description of the development and initial validation of the tool are presented in the previous work by Ewer et al. [25]. Face and content validity was established with adults and adolescents and convergent and known-group validity was assessed in adolescents with HSD/hEDS [25].

### Data management and analysis

The IBM® SPSS® package version 29 was used for descriptive and inferential statistics calculations. Only complete data were analysed. Partially completed questionnaires were omitted from the data analysis. Convergent and known-group validity analyses were used to assess construct validity of each Spider domain. Normality of data was assessed through visual inspection of the histograms and Shapiro–Wilk test. Between-group differences were analysed using chi-square test and independent *t*-tests for categoric data or Mann–Whitney *U* for numeric data. Convergent validity was assessed through Spearman’s Rho analysis using data collected from the SH group. Convergent validity is deemed unacceptable if correlations are below *r* = 0.50, correlations of *r* > 0.6 are considered moderate, and *r* > 0.7 are considered strong [42]. A *p*-value of < 0.05 was considered statistically significant, and bootstrapping was used to calculate 95% confidence intervals for non-parametric tests. Known-group validity was calculated by comparing scores of the Spider domains from the SH and healthy control groups. Mann–Whitney *U* tests were used due to non-parametric data.

## Results

### Participants

**Fig. 2:**
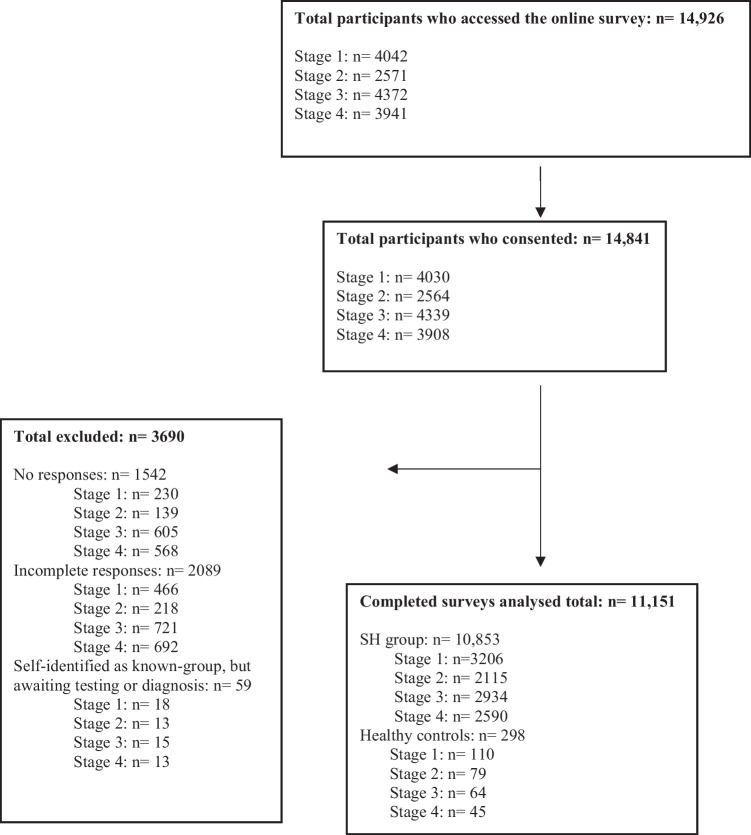
Participant recruitment flow diagram. Descriptive caption: a flow diagram showing the stages of recruitment from participants accessing the questionnaire, consenting (14,841) and completing the survey (11,151). Three thousand six hundred ninety participants were excluded if their responses were missing or incomplete or if they self-identified as the control group but reported symptomatic hypermobility

Across the four stages, there were a total of 11,151 participants included from an initial cohort of 14,926. There were 10,853 participants with SH and 298 healthy controls. A Prisma flow diagram (Fig. 2) shows the recruitment flow, including participant numbers across all four stages and per individual stage.

Demographic data are presented in Table 2. Across all stages, most patients were white, female, and from the United Kingdom (UK) or North America. In stages 1 to 4, there were 3206, 2115, 2934, and 2590 participants recruited in the SH group and 110, 79, 64, and 45 health controls respectively. There were significant between-group differences in sex and gender across all stages. In stage one, there were between-group differences in country of residence, and in stages 1, 2, and 4, there were also between-group differences in age.

**Table 2:** Demographic characteristics of participants in each stage

Demographic characteristics		Stage 1			Stage 2			Stage 3			Stage 4		
		SH *n* = 3206	Controls *n* = 110	Significance *p*-value	SH *n* = 2115	Controls *n* = 79	Significance *p*-value	SH *n* = 2934	Controls *n* = 64	Significance *p*-value	SH *n* = 2590	Controls *n* = 45	Significance *p*-value
Age (years)^†^		38.62 ± 11.73 38 (19)	40.88 ± 12.12 42 (20)	0.047*	38.32 ± 11.80 38 (18)	43.03 ± 12.56 43 (24)	0.001*	37.05 ± 11.74 36 (19)	39.72 ± 13.93 40 (24)	0.152	36.83 ± 11.73 36 (18)	41.42 ± 13.94 43 (25)	0.022*
Sex**	Female	3110 (97%)	81 (73.6%)	< 0.001*	2060 (97.4%)	68 (86.1%)	< 0.001*	2849 (97.1%)	54 (84.4%)	< 0.001*	2515 (97.1%)	39 (86.7%)	0.003*
	Intersex	5 (0.2%)	1 (0.9%)		1 (0.1%)	0		8 (0.3%)	0		7 (0.3%)	0	
	Male	91 (2.8%)	28 (25.5%)		54 (2.5%)	11 (13.9%)		77 (2.6%)	10 (15.6%)		68 (2.6%)	6 (13.3%)	
Gender**	Woman	2828 (88.2%)	72 (65.5%)	< 0.001*	1842 (87.1%)	62 (78.5%)	0.016*	2494 (85%)	43 (67.2%)	< 0.001*	2198 (84.9%)	30 (66.7%)	0.003*
	Man	123 (3.8%)	30 (27.3%)		74 (3.5%)	10 (12.7%)		120 (4.1%)	9 (14.1%)		107 (4.1%)	6 (13.3%)	
	Non-binary	208 (6.5%)	5 (4.5%)		150 (7.1%)	7 (8.9%)		253 (8.6%)	9 (14.1%)		222 (8.6%)	6 (13.3%)	
	A gender not listed here	6 (0.2%)	2 (1.8%)		10 (0.5%)	0		12 (0.4%)	1 (1.6%)		11 (0.4%)	1 (2.2%)	
	Unsure	34 (1.1%)	1 (0.9%)		30 (1.4%)	0		42 (1.4%)	1 (1.6%)		40 (1.5%)	1 (2.2%)	
	Prefer not to say	7 (0.2%)	0		9 (0.4%)	0		13 (0.4%)	1 (1.6%)		12 (0.5%)	1 (2.2%)	
Gender same as sex?**	Yes	2950 (92%)	101 (91.8%)	0.643	1920 (90.8%)	73 (92.4%)	0.777	2613 (89.1%)	52 (81.3%)	0.081	2301 (88.8%)	37 (82.2%)	0.126
	No	225 (7%)	9 (8.2%)		159 (7.5%)	6 (7.6%)		274 (9.3%)	10 (15.6%)		245 (9.5%)	6 (13.3%)	
	Prefer not to say	31 (1%)	0		36 (1.7%)	0		47 (1.6%)	2 (3.1%)		44 (1.7%)	2 (4.4%)	
Ethnicity**	Asian/Asian British	23 (0.7%)	1 (0.9%)	0.885	18 (0.9%)	1 (1.3%)	0.270	21 (0.7%)	0	0.086	19 (0.7%)	0	0.120
	Black/African/Caribbean/Black British	16 (0.5%)	0		7 (0.3%)	1 (1.3%)		8 (0.3%)	0		8 (0.3%)	0	
	Mixed/multiple Ethnicity	192 (6%)	8 (7.3%)		125 (5.9%)	3 (3.8%)		209 (7.1%)	2 (3.1%)		180 (6.9%)	2 (4.4%)	
	Others	87 (2.7%)	3 (2.7%)		47 (2.2%)	3 (2.8%)		87 (3%)	3 (4.7%)		83 (3.2%)	1 (1.7%)	
	White	2886 (90%)	98 (89.1%)		1917 (90.6%)	71 (89.9%)		2608 (88.9%)	58 (90.6%)		2299 (88.8%)	40 (88.9%)	
	Prefer not to say	2 (0.1%)	0		1	0		1 (0.0%)	1 (1.6%)		1 (0.0%)	1 (2.2%)	
Country of residence**	Africa	9 (0.3%)	0		8 (0.4%)	1 (1.3%)		11 (0.4%)	0		11 (0.4%)	0	
	Asia	20 (0.6%)	1 (0.9%)	0.046*	7 (0.3%)	0	0.133	9 (0.3%)	0	0.683	9 (0.3%)	0	0.627
	Australia/Oceania	162 (5.1%)	9 (8.2%)		122 (5.8%)	6 (7.6%)		177 (6%)	6 (9.4%)		140 (5.4%)	2 (4.4%)	
	Europe	231 (7.2%)	12 (10.9%)		144 (6.8%)	4 (5.1%)		235 (8%)	6 (9.4%)		211 (8.1%)	2 (4.4%)	
	North America and Canada	1114 (35.7%)	45 (40.9%)		887 (41.9%)	40 (50.6%)		1456 (49.6%)	29 (45.3%)		1290 (49.8%)	21 (46.7%)	
	Republic of Ireland	24 (0.7%)	1 (0.9%)		18 (0.9%)	1 (1.3%)		21 (0.7%)	1 (1.6%)		20 (0.8%)	1 (2.2%)	
	South America	35 (1.1%)	3 (2.7%)		18 (0.9%)	2 (2.5%)		39 (1.3%)	1 (1.6%)		34 (1.3%)	1 (2.2%)	
	UK	1581 (49.3%)	39 (35.5%)		911 (43.1%)	25 (31.6%)		986 (33.6%)	21 (32.8%)		875 (33.8%)	18 (40%)	

Key: ^†^numeric data displayed as mean ± standard deviation or median (interquartile ranges). **All other categorical data displayed as counts and frequencies in percentages (%). *Significance level *p* < 0·05. Abbreviations: *n* sample size, *SH* symptomatic hypermobility group

### Construct validity

Convergent validity results are shown in Table 3. All results were statistically significant (*p* < 0.001). Strong positive correlations were found between the Spider neuromusculoskeletal domain and select questions of the BIoH questionnaire (*r* = 0.73), between the Spider gastrointestinal domain and the GSRS questionnaire (*r* = 0.80), and between the Spider cardiac domain and the COMPASS-31 (*r* = 0.74). Strong correlations were also demonstrated between the Spider anxiety and depression domains and the GAD-7 and PHQ-9 (*r* = 0.73 and *r* = 0.79) respectively and between the Spider urogenital domain and the LURN- SI-29 (*r* = 0.78). Moderate positive correlations were found between the Spider fatigue domain and the combined scores from the CIS and PSQI (*r* = 0.63) and between the Spider anxiety and depression domains and the HADS anxiety and depression questions respectively (*r* = 0.67 and *r* = 0.69) with 300 of the participants. Moderate positive correlations were also found between the Spider pain domain and select questions from the MPI and BIoH (*r* = 0.62 and *r* = 0.68 respectively).

**Table 3:** Convergent validity of each Spider domain in the whole SH sample

The Spider domain	Correlated with PROMs	Spearman’s correlation (*r*)	95% CI**	*p*-value
Neuromusculoskeletal	BIoH	0.73	0.71 to 0.74	< 0.001*
Fatigue	PSQI + CIS	0.63	0.60 to 0.65	< 0.001*
Gastrointestinal	GSRS	0.80	0.78 to 0.81	< 0.001*
Anxiety	GAD-7	0.73	0.71 to 0.76	< 0.001*
	HADS-A	0.67	0.60 to 0.74	< 0.001*
Depression	PHQ-9	0.79	0.77 to 0.81	< 0.001*
	HADS-D	0.69	0.62 to 0.75	< 0.001*
Cardiac dysautonomia	COMPASS-31	0.74	0.72 to 0.76	< 0.001*
Pain	MPI	0.62	0.59 to 0.64	< 0.001*
	BIoH	0.68	0.65 to 0.70	< 0.001*
Urogenital	LURN-SI-29	0.78	0.76 to 0.80	< 0.001*

Key: *BIoH* Bristol Impact of Hypermobility questionnaire, *CI* confidence interval, *CIS* Checklist of Individual Strengths, *GAD-7* Generalised Anxiety Disorder, *GSRS* Gastrointestinal Symptom Rating Scale, *HADS-A* Hospital Anxiety and Depression Scale anxiety questions, *HADS-D* Hospital Anxiety and Depression Scale depression questions, *LURN-SI-29* LURN Symptom Index-29, *MPI* Multidimensional Pain Inventory, *PHQ-9* Patient Health Questionnaire. *Significance level at *p* < 0.05. **Confidence intervals presented as lower limit to upper limit. Confidence intervals calculated through bootstrapping in SPSS

**Fig. 3:**
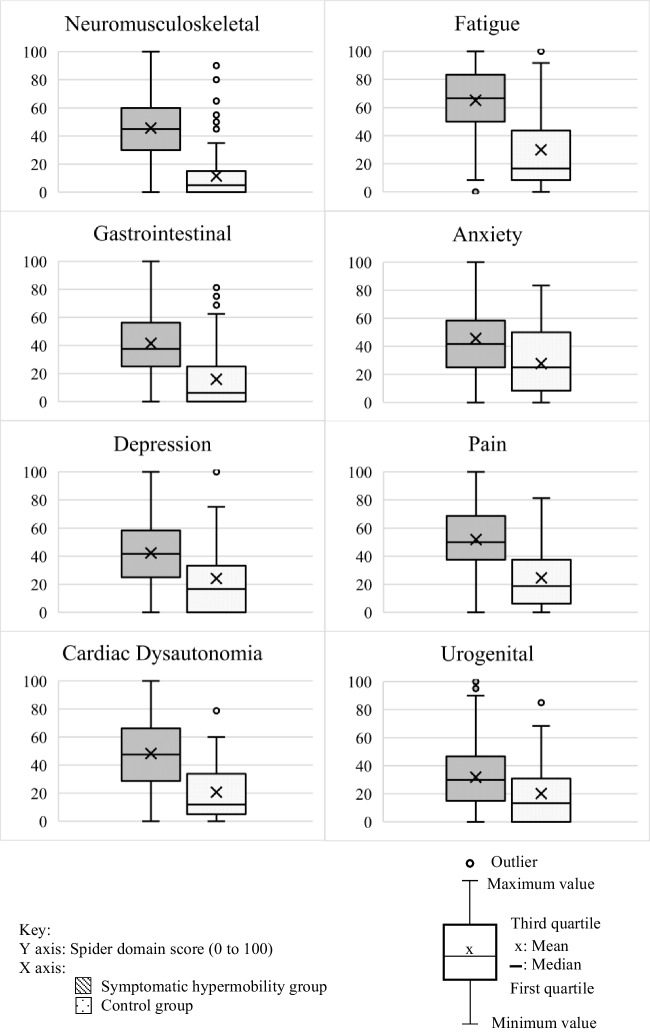
Box and whisker plots demonstrating differences between domain scores of SH and control groups. Descriptive caption: A figure showing eight box and whisker plots for each domain of the Spider questionnaire. The graph shows the difference between Spider domain scores of the symptomatic hypermobile group and the control group. The whiskers show the minimum and maximum values, the box shows the third quartile as the top line, the median as a line across the middle, and first quartile as a line at the bottom. The mean is represented by a cross, and outliers are represented by circles

Known-group and descriptive statistics are shown in Table 4, and box and whisker plots showing the known-group analysis are shown in Fig. 3. There were statistically significant differences in all Spider domain scores between participants with SH and non-hypermobile controls (*p* < 0.001). Data is presented as mean (SD) or median (IQR) depending on normality. Large differences were found between the SH and control groups in the neuromusculoskeletal domain: 45.65 (SD 19.66) and 5 (IQR 0–15), gastrointestinal domain: 41.48 (SD 22.17) and 6.25 (IQR 0–25), and fatigue domain: 66.67 (IQR 50–83.33) and 16 (IQR 8.3–43.75) respectively. Large differences were also seen between the SH and control groups in the depression domain: 41.67 (IQR 25–58.33) and 16.67 (IQR 0–33.33), cardiac domain: 47.5 (IQR 28.75–66.25) and 11.8 (IQR 5–33.75), and pain domain: 51.93 (SD 20.37) and 18.75 (IQR 6.25–37.5). Moderate differences were found between the SH and control groups in the anxiety domain: 45.55 (SD 23.51) and 25 (IQR 8.33–50) and the urogenital domain: 30 (IQR 15.46.67) and 13.33 (IQR 0–30.83).

**Table 4:** Known-group validity and descriptive statistics

The Spider domain	Descriptive statistics SH-group^†^		Descriptive statistics control group^†^		Mann–Whitney *U*	*p*-value
	Mean (SD)	Median (Q1–Q3)	Mean (SD)	Median (Q1–Q3)		
NMSK	*n* = 3206		*n* = 110			
	45.65 ± 19.66	45 (30–60)	11.5 ± 17.75	5 (0–15)	32,617.5	< 0.001*
Fatigue	*n* = 3206		*n* = 110			
	65.22 ± 21.53	66.67 (50–83.33)	29.92 ± 28.45	16 (8.3–43.75)	62,705.5	< 0.001*
Gastrointestinal	*n* = 3206		*n* = 110			
	41.48 ± 22.17	37.5 (25–56.25)	15.91 ± 19.79	6.25 (0–25)	63,129	< 0.001*
Anxiety	*n* = 2115		*n* = 79			
	45.55 ± 23.51	41.67 (25–58.33)	27.75 ± 24.63	25 (8.33–50)	49,611.5	< 0.001*
Depression	*n* = 2115		*n* = 79			
	42.33 ± 26.63	41.67 (25–58.33)	24.16 ± 26.77	16.67 (0–33.33)	49,162.5	< 0.001*
Cardiac dysautonomia	*n* = 2934		*n* = 64			
	48.22 ± 24.69	47.5 (28.75–66.25)	20.72 ± 20.67	11.8 (5–33.75)	37,387	< 0.001*
Pain	*n* = 2934		*n* = 64			
	51.93 ± 20.37	50 (37.5–68.75)	24.61 ± 22.71	18.75 (6.25–37.5)	35,694.5	< 0.001*
Urogenital	*n* = 2590		*n* = 45			
	31.86 ± 21.13	30 (15–46.67)	20.15 ± 22.25	13.33 (0–30.83)	37,311	< 0.001*

Key: *IRQ* interquartile ranges, *n* number of participants, *NMSK* neuromusculoskeletal, *SD* standard deviation, SH symptomatic hypermobility. ^†^Numeric data displayed as mean ± standard deviation and median (first quartile to third quartile). *Significance level at *p* < 0.05

### Sub-group analysis

The authors recognised that the sample was predominantly white participants, which may not reflect the heterogeneity of the HSD/hEDS population. A secondary analysis was completed excluding white participants to assess the impact of ethnicity on the convergent validity results. The domains retained similar statistically significant (*p* < 0.001) moderate-to-strong correlations. In stage 1, 320 participants self-identifying as Black, Asian, mixed or multiple, or other ethnicities were included. In the NMSK, fatigue, and gastrointestinal domains, correlations of *r* = 0.71, *r* = 0.65, and *r* = 79 were found with the BIoH, CIS + PSQI, and the GSRS respectively. In stage 2, 198 participants were included in the sub-group analysis and correlations of *r* = 0.75 and *r* = 0.79 were found between the Spider anxiety and depression domains and the GAD-7 and PHQ-9. In stage 3, 326 participants were included, with correlations of *r* = 0.72, *r* = 0.72, and *r* = 0.69 found between the Spider cardiac domains and the COMPASS-31 and the Spider pain domain the MPI and the BIoH respectively. In stage 4, 291 participants were included in the sub-group analysis. A strong correlation of *r* = 0.76 was found between the Spider urogenital domain and the LURN-SI-29.

## Discussion

This paper is the second in a series validating the Spider questionnaire, a novel, hypermobility-specific tool whose results provide insight into the multisystemic symptom profile of individuals with HSD/hEDS. This study found acceptable convergent and known-group validity for all Spider domains in a population of adults with SH. The results are in keeping with the previous validation study with adolescents, which found acceptable convergent validity in all domains and known-group validity in seven domains [25]. When correlating the Spider domains with validated, appropriate comparator questionnaires, the 95% confidence intervals remained above the recommended correlation of *r* > 0·5 in all domains [43]. These studies collectively demonstrate that the Spider can accurately capture the concept of the multisystemic symptoms/comorbidities in individuals aged 13 to 65 and in most domains distinguish between individuals with and without SH.

### Strengths of the Spider

To the best of our knowledge, the Spider questionnaire is the first disease-specific tool to provide a broad, visual overview of the presence and impact of multisystemic symptoms and comorbidities that have consistently been associated with JH. The Spider is concise, capturing a broad clinical overview through only 31 questions, which were developed collaboratively by specialist clinicians, researchers, and patients. The questionnaire takes around 10 min to complete, without the need for multiple, time-consuming, and lengthy PROMs. This reduces the burden on both patients and clinicians and can be used without compromising patient energy or appointment time, which is particularly limited in government healthcare. It ensures that the individual is assessed holistically and that the non-musculoskeletal symptoms, which are often neglected during assessment, are considered when deciding multidisciplinary management. There is a lack of recognition of the multisystemic nature of HSD/hEDS, leading to unmet medical needs and impacting QoL greatly [24]. The Spider can be used by any member of the multidisciplinary team to guide the conversation between patients and clinicians, ensuring the presence and impact of multiple comorbid symptoms and conditions are considered when deciding and prioritising care. The Spider can also be completed independently by patients, which will help them in voicing their concerns and management priorities succinctly during healthcare appointments. Upon completion, a radar graph providing a visual overview of the symptom profile is created, which is easy to interpret.

### Study strengths and limitations

#### Sampling procedure

As with the adolescent study, the use of international charities during recruitment resulted in a very large sample of international participants. Over 11,000 participants from various locations and ethnicities participated in total, with 2000 to 3000 participants per stage. Whilst there are often concerns with large sample sizes, such as increased study costs and researcher burden, syntax formulas created prior to data collection were used to complete data analysis without increasing analysis time. The larger sample size increases the likelihood that the study sample represents the HSD/hEDS population and improves generalisability of results. As with previous studies, the sample was largely white and female. Whilst HSD/hEDS are more common in females, the disparity is larger than reported in the literature [44]. The choice of sampling through self-selection may have introduced bias and limited the ethnic diversity of the sample. Researchers also encouraged snowball sampling, which may have increased homogeneity of the sample through referrals to friends or family with similar demographic characteristics. However, this recruitment did allow for inclusion of a broad, international participant sample which would not have been practical or feasible with other recruitment methods. To mitigate the impact of sample homogeneity on the results, a secondary sub-group analysis excluding white participants was completed and showed similar convergent validity correlations to the whole sample analysis. This suggests the findings of this research are still generalisable to other ethnic groups, despite the reduced representation in this sample.

#### Validation procedure

To establish convergent validity, comparator questionnaires measuring similar concepts are used and results are compared using correlational analysis. Whilst this is an accepted method of establishing validity, it must be acknowledged that there are currently no gold standard PROMs recommended as a comparator in hypermobile patient populations [45]. The choice of comparator questionnaires was guided by the recommendations and the Common Data Elements developed by the Ehlers-Danlos Society Internal Consortium, expertise of researchers and clinicians specialising in SH, and assessment of the psychometric properties of available PROMs [46]. As the nature of SH is complex, diverse, and varied, the research team used multiple measures to ensure all concepts measured in the Spider domains were captured. Instead of the full MPI, BIoH, and COMPASS-31 questionnaires, specific questions were selected to validate the pain, neuromusculoskeletal, and cardiac dysautonomia domains respectively. This decision was made due to the lack of validated comparator questionnaires which captured the specific symptoms that hypermobile patients present. In several domains, multiple PROMs were used as comparators. In the pain domain, the MPI sections A and C were used as a general measure of overall pain but select questions from the BIoH were required to assess the more specific symptoms associated with hypermobility. In the Spider anxiety and depression domain validation, the HADS questionnaire was used in conjunction with the GAD-7 and PHQ-9. The HADS has been used widely in hypermobility research, but as there are licensing costs, study funding could only accommodate its use with 300 participants. Large participant numbers were expected, and researchers did not want to limit the assessment of the anxiety and depression domains, so the GAD-7 and PHQ-9 were used as additional comparators. Whilst these are less commonly used in hypermobility research, they are validated with strong psychometric properties in other chronic health conditions [47, 48]. Finally, in the fatigue domain, the CIS was the preferred comparator, being widely used in hypermobility research [49–51], but it did not contain a question about sleep. As such, the PSQI was added to evaluate sleep quality and fatigue and the scores combined with the CIS.

### Developments and further research

A cost-free digital application is being developed which can be accessed by clinicians and researchers internationally. It is anticipated that the Spider will allow monitoring of the natural history and interindividual variability of the multisystemic comorbidities associated with HSD/hEDS. This may enable future studies to identify determinates that influence prognosis and highlight treatment priorities. Future research possibilities include reliability analysis, including internal consistency, test–retest reliability, and factor structure analysis with adolescents and adults. Following this, minimal clinically important differences will be established so the Spider can be used to track meaningful symptom change and assess the effects of treatment. The Spider could be used to monitor the effect of multidisciplinary interventions and rehabilitation programmes on the whole symptom profile. The Spider will be translated using forward-backwards translation to ensure it is accessible internationally. Finally, the research team predict the Spider has the potential to be developed with other heritable connective tissue disorders.

## Conclusion

This study adds to a body of work establishing psychometric properties of the Spider, a unique and symptom-specific multisystemic questionnaire for people with SH. Statistically significant, moderate-to-strong convergent validity and statistically significant known-group validity were shown in all questionnaire domains. The Spider is a novel, concise, efficient, and valid tool, freely accessible to clinicians and researchers to assess the multisystemic symptom profiles of people aged 13 to 65 with SH. The questionnaire enables clinicians to ensure patient care is individualised, holistic, and tailored, to meet the specific needs of the individual with SH.

## Data Availability

Authors had full control over all primary data. The datasets generated during and/or analysed during the current study are available from the corresponding author upon reasonable request.

## Declarations

### Permission to reproduce material from other sources

Permission was granted for use of all comparator outcome measures by the creators of the tools for use within this research.
